# Modelling multiple time-scales with flexible parametric survival models

**DOI:** 10.1186/s12874-022-01773-9

**Published:** 2022-11-09

**Authors:** Nurgul Batyrbekova, Hannah Bower, Paul W. Dickman, Anna Ravn Landtblom, Malin Hultcrantz, Robert Szulkin, Paul C. Lambert, Therese M-L. Andersson

**Affiliations:** 1 Department of Medical Epidemiology and Biostatistics, Karolinska Institutet, Stockholm, Sweden; 2 SDS Life Science AB, Stockholm, Sweden; 3 Clinical Epidemiology Division, Department of Medicine, Solna, Karolinska Institutet, Stockholm, Sweden; 4 Department of Medicine, Solna, Karolinska Institutet, Stockholm, Sweden; 5 Department of Medicine, Division of Hematology, Stockholm South Hospital, Stockholm, Sweden; 6 Department of Medicine, Myeloma Service, Memorial Sloan-Kettering Cancer Center, New York, NY USA; 7 Biostatistics Research Group, Department of Health Sciences, University of Leicester, Leicester, UK

**Keywords:** Multiple time-scales, Flexible parametric survival models, Time-varying covariate, Matched cohort, Cohort studies, Epidemiological methods

## Abstract

**Background:**

There are situations when we need to model multiple time-scales in survival analysis. A usual approach in this setting would involve fitting Cox or Poisson models to a time-split dataset. However, this leads to large datasets and can be computationally intensive when model fitting, especially if interest lies in displaying how the estimated hazard rate or survival change along multiple time-scales continuously.

**Methods:**

We propose to use flexible parametric survival models on the log hazard scale as an alternative method when modelling data with multiple time-scales. By choosing one of the time-scales as reference, and rewriting other time-scales as a function of this reference time-scale, users can avoid time-splitting of the data.

**Result:**

Through case-studies we demonstrate the usefulness of this method and provide examples of graphical representations of estimated hazard rates and survival proportions. The model gives nearly identical results to using a Poisson model, without requiring time-splitting.

**Conclusion:**

Flexible parametric survival models are a powerful tool for modelling multiple time-scales. This method does not require splitting the data into small time-intervals, and therefore saves time, helps avoid technological limitations and reduces room for error.

**Supplementary Information:**

The online version contains supplementary material available at 10.1186/s12874-022-01773-9.

## Introduction

In survival analysis, the event rates may depend on multiple time-scales simultaneously, such as time-on-study, attained age, time since disease onset, etc, all with different time-origins, such as start date of the study, birth, onset of disease, etc. To fit survival models with multiple time-scales, it is standard practice to split the data into short time-intervals along the relevant time-scales, and fit a Cox model or a Poisson generalised linear model with categories for the time-intervals [1]. Using the Cox model may not be the best approach if we are interested in modelling the rates over the multiple time-scales, since the baseline hazard function is not estimated in the Cox model, and one of the time-scales has to be selected as the baseline [2]. Furthermore, fitting either the Cox or Poisson model to the time-split data is based on the assumption of piecewise constant hazard rates within the time-intervals. Fractional polynomials [3] or splines [4] can be used instead to obtain smooth estimates of the baseline hazard function in the Poisson model. Even so, this requires splitting the data into short intervals which often leads to very large datasets, thus adding to the challenges of fitting computationally burdensome models.

In this article, we propose using flexible parametric survival models (FPMs) [5, 6] as an alternative approach when fitting hazard models with multiple time-scales. This method allows for modelling multiple time-scales as continuous functions and does not require time-splitting. FPMs with two time-scales are presented through two scenarios. In the first scenario, the second time-scale is introduced when individuals experience an intermediate event, which is considered as a time-varying covariate. In the second scenario, both time-scales are present from the start of the study but the second time-scale is relevant only for a subset of individuals in the matched cohort. We also compare the results to those from fitting Poisson models. The choice of the optimal single time-scale or how to combine multiple time-scales into one time-scale has been widely discussed [7–12] and is not the focus of this study.

## Notation and background

### Proportional hazards models with multiple time-scales

Modelling hazard rates in terms of multiple time-scales has been described in different settings, for example in age, period and cohort models [13] and in the Lexis model with two time-axes [14], and the two-way proportional hazards (PH) model [15]. A thorough description of the general approach to modelling multiple time-scales within multistate models using Poisson framework has been provided by Iacobelli and Carstensen (2013) [16].

In a model with two time-scales and proportional hazards, i.e. a model where time-scales are modelled as main effects, the log hazard function can be expressed as:

1 $$\begin{aligned} \log (h(t_1, t_2; \boldsymbol{\gamma }_{p}, \boldsymbol{\gamma }_{s}, \boldsymbol{\beta })) = p_0(t_1; \boldsymbol{\gamma }_{p}) + s_0(t_2; \boldsymbol{\gamma }_{s}) + \boldsymbol{x}\boldsymbol{\beta }, \end{aligned}$$

where $p_0$ and $s_0$ are the baseline hazard functions (which can be any functions) for time-scales $t_1$ and $t_2$, respectively, with $\boldsymbol{\gamma }_{p}$ and $\boldsymbol{\gamma }_{s}$ as the corresponding parameter vectors. An intercept is included in one of the two functions. The covariates of interest are expressed as $\boldsymbol{x}$ with associated log hazard ratios $\boldsymbol{\beta }$.

Provided chosen time-scales are measured in the same units of time, and their time-origins are known then the time-scales can be expressed as functions of one reference time-scale and corresponding offset terms. For example, if $t_1$ is time since diagnosis and $t_2$ is attained age then the time-origin for $t_1$ is diagnosis and for $t_2$ it is birth. Then using age at diagnosis, $a_0$, as an offset, we can write $t_2 = t_1 + a_0$, or symmetrically $t_1 = t_2 - a_0$. Thus, with $t_1$ as the reference time-scale, model (1) can be written as,

2 $$\begin{aligned} \log (h(t_1, t_2; \boldsymbol{\gamma }_{p}, \boldsymbol{\gamma }_{s}, \boldsymbol{\beta }))= & {} p_0(t_1; \boldsymbol{\gamma }_{p}) + s_0(t_2; \boldsymbol{\gamma }_{s}) + \boldsymbol{x}\boldsymbol{\beta }\nonumber \\= & {} p_0(t_1; \boldsymbol{\gamma }_{p}) + s_0(t_1 + a_0; \boldsymbol{\gamma }_{s}) + \boldsymbol{x}\boldsymbol{\beta } \end{aligned}$$

Additionally, if a third time-scale of interest is the calendar time, $t_3$ (origin 0 CE), then we can write it as a function of $t_1$, as $t_3 = t_1 + c_0$, or as a function of $t_2$ as $t_3 = t_2 - a_0 + c_0$, where $c_0$ is the calendar date of diagnosis and acts as another offset term. To demonstrate further the symmetry of model (2) with additional time-scale, we can express the model with three time-scales $t_1, t_2, t_3$ as a function of the reference time-scale $t_2$ and offset terms $a_0, c_0$,

3 $$\begin{aligned} \log (h(t_1, t_2, t_3; \boldsymbol{\gamma }_{p}, \boldsymbol{\gamma }_{s}, \boldsymbol{\gamma }_{q}, \boldsymbol{\beta }))= & {} p_0(t_1; \boldsymbol{\gamma }_{p}) + s_0(t_2; \boldsymbol{\gamma }_{s}) + q_0(t_3; \boldsymbol{\gamma }_{q}) + \boldsymbol{x}\boldsymbol{\beta } \nonumber \\= & {} p_0(t_2 - a_0; \boldsymbol{\gamma }_{p}) + s_0(t_2; \boldsymbol{\gamma }_{s}) + q_0(t_2 - a_0 + c_0; \boldsymbol{\gamma }_{q}) + \boldsymbol{x}\boldsymbol{\beta } \end{aligned}$$

#### Non-proportional hazards models with multiple time-scales

Models (1) - (3) can be extended further to include non-proportional hazards, i.e. interactions between covariates and one or more of the time-scales. For example, model (1) with interaction between covariates $x_l$ ($l=1,\dots , L$) and time-scale $t_1$ is written as:

4 $$\begin{aligned} \log (h(t_1, t_2; \boldsymbol{\gamma }_{p}, \boldsymbol{\gamma }_{s}, \boldsymbol{\beta })) = p_0(t_1; \boldsymbol{\gamma }_{p}) + s_0(t_2; \boldsymbol{\gamma }_{s}) + \boldsymbol{x}\boldsymbol{\beta } + \Sigma ^{L}_{l=1}x_l p_{l}(t_1; \boldsymbol{\gamma }_l) \end{aligned}$$

The covariates included in $\varvec{x}$ can be of any functional form and therefore, in model (4) it is not necessarily a linear form that is used in the interaction term with the time-scale. Additionally, interactions between time-scales can also be included, which means that the effect of the first time-scale on the outcome can differ along the second time-scale. In further discussions and examples, we will focus on fitting PH models with two time-scales using flexible parametric survival models and compare them to Poisson models.

### Flexible parametric survival models with multiple time-scales

Flexible parametric survival models (FPMs) were introduced by Royston and Parmar (2002) [5] and have been further developed by others [6, 17, 18]. FPMs on the log-hazard scale use a smoothing function for the baseline hazard in a form of restricted cubic splines, which are piecewise cubic polynomial functions that are joined at pre-specified positions (knots). The restricted cubic splines are often created on the log-scale of time. Users are required to choose a number of knots for the splines, as well as the position of the knots. Sensitivity analyses have shown that estimates produced by FPMs are robust to the number and placement of knots [19].

As described in Section 2.1, if the time-scales of interest can be expressed in terms of one time-scale and the offset terms then, in its simplest form, the PH FPM on the log hazard scale with two time-scales, $t_1$ and $t_2$ is written as

5 $$\begin{aligned} \log (h(t_1, t_2; \boldsymbol{\gamma }_{p}, \boldsymbol{k}_{p}, \boldsymbol{\gamma }_{s}, \boldsymbol{k}_{s}, \boldsymbol{\beta })) = p_0(t_1; \boldsymbol{\gamma }_{p}, \boldsymbol{k}_{p}) + s_0(t_2; \boldsymbol{\gamma }_{s}, \boldsymbol{k}_{s}) + \boldsymbol{x}\boldsymbol{\beta }, \end{aligned}$$

where the baseline hazard functions on the $t_1$ and $t_2$ time-scales are represented by the restricted cubic spline functions, $p_0(t_1; \boldsymbol{\gamma }_{p}, \boldsymbol{k}_{p})$ and $s_0(t_2; \boldsymbol{\gamma }_{s}, \boldsymbol{k}_{s})$, respectively. The knot location vectors, $\boldsymbol{k}_{p}$ and $\boldsymbol{k}_{s}$ are either chosen by default at equally spaced centiles over the distribution of the event-times or determined by the user. Also this model can be easily extended to include interactions between the time-scales as well as non-proportional hazards for the covariates of interest.

### Poisson model with multiple time-scales

We can also use the Poisson approach to fit the same underlying rate models described in Section 2.1 since the likelihood of the rate model is equivalent to the likelihood of a Poisson model [15, 16, 20]. To do this, users are required to split the data along the relevant time-scales into intervals short enough to better support the assumption of constant hazard rates within the intervals, which can come at the cost of high computational burden. Depending on the research area, the data can be split along one time-scale, and the other time-scale is obtained using the offset between time-scales. However, in some situations splitting is performed along two or more time-scales. The baseline hazard functions for the time-scales can be either piecewise constant, or smooth functions based on the intervals (for example, the start or the mid-point of each interval). The log of person-time within each interval is included in the model as an offset.

For consistency with the FPM approach, in this study we use restricted cubic splines when using the Poisson approach to estimate the smooth effect of the time-scales on the baseline hazard.

Non-proportional hazards as shown by model (4) as well as interactions between time-scales can also be modelled within the Poisson framework.

## Application scenarios

To illustrate models with multiple time-scales we analysed two different case-studies, using both FPMs and Poisson models. The models displayed are essentially the same, however they illustrate different scenarios where multiple time-scales can arise. The first case-study includes a time-varying variable that introduces a second time-scale upon changing its value, and the second is a matched cohort study where the second time-scale is relevant only for the exposed subjects. In both case-studies our objective was to model mortality rates with two time-scales, and graphically represent the estimated rates and the survival proportions over different time-points on both time-scales.

### Time-varying covariate

Similar to the illness-death example discussed by Iacobelli and Carstensen (2013) [16], for modelling multiple time-scales, we analysed cohort data of 2,982 individuals diagnosed during 1978-1993 with primary breast cancer in Rotterdam, who had undergone primary surgery [21, 22]. Patients were followed from the initial state *surgery* until the event of interest *death* or censoring at 10 years post surgery. Throughout the follow-up it is also known whether and when they experienced the intermediate state *relapse or metastasis* (RM). RM can, therefore, be treated as a time-varying covariate, and time since RM as a secondary time-scale.

In total 1,139 patients died during the total follow-up of 19,937 person-years (overall mortality rate 57 per 1000 person-years), and 1,004 of these deaths occurred after RM. During follow-up, 1,477 patients experienced RM, and the median time to RM among those with RM was 2.43 years (min = 0.1, max = 9.99).

Using the FPM and the Poisson methods, we fitted the following hazard model with two time-scales, $t_1$ as time since surgery (reference time-scale) and $t_1 - r$ as time since RM with *r* as the time of RM,

6 $$\begin{aligned} \log (h(t_1, t_1-r; \boldsymbol{\gamma }_{p}, \boldsymbol{k}_{p}, \boldsymbol{\gamma }_{s}, \boldsymbol{k}_{s}, \boldsymbol{\gamma }_{q}, \boldsymbol{k}_{q}, \boldsymbol{\beta }))= & {} p_0(t_1; \boldsymbol{\gamma }_{p}, \boldsymbol{k}_{p}) + s_0(t_1-r; \boldsymbol{\gamma }_{s}, \boldsymbol{k}_{s}) \cdot I_{RM} \nonumber \\ &+ \beta _{RM} \cdot I_{RM} + q(age; \boldsymbol{\gamma }_{q}, \boldsymbol{k}_{q}) + \beta _{horm} \cdot I_{horm}, \end{aligned}$$

where $I_{RM}$ was used as a time-varying indicator for patients with RM, so that the effect of time since RM on the baseline hazard can only be estimated after patients have transitioned to RM. In both methods, for the restricted cubic spline functions, $p_0, s_0$, we chose four knots equally placed over the distribution of event-times, and we used the same knots in both methods, however, in $p_0$ we created splines for $\log (t_1)$. An intercept of the model is included in function $p_0$ as part of the parameters for the reference time-scale $t_1$. As in the model above $s_0$ does not include an intercept. There is a choice to be made whether to include a parameter for an immediate change in the hazard at the time of RM, and setting $\beta _{RM}$ to 0 would remove this change. We also included in the model a restricted cubic spline function of *age* (age at surgery) with six knots and an indicator $I_{horm}$ for hormonal therapy, assuming proportional hazards for these covariates over both time-scales by not including interactions with either of the time-scales.

To fit this model, it was necessary to create two rows per individuals for those that experienced RM, where indicator $I_{RM}$ changed from 0 to 1 at the time of RM. Furthermore, for the Poisson approach, we split the reference time-scale, time since surgery, into two-day-intervals, and used mid-points of the intervals for creating the splines. For this cohort it may be unnecessary to have such short intervals, especially in later part of the follow-up, but for other diseases it may be essential.

For the purpose of this study, we chose to keep the model simple in terms of the number of covariates, and we did not focus on the question of model-specification. Therefore, the results may not be clinically representative.

The implementation of analyses for the FPM method for this cohort using Stata code is provided in the Supplementary material.

#### Graphical representations of results for model with a time-varying covariate

**Fig. 1:**
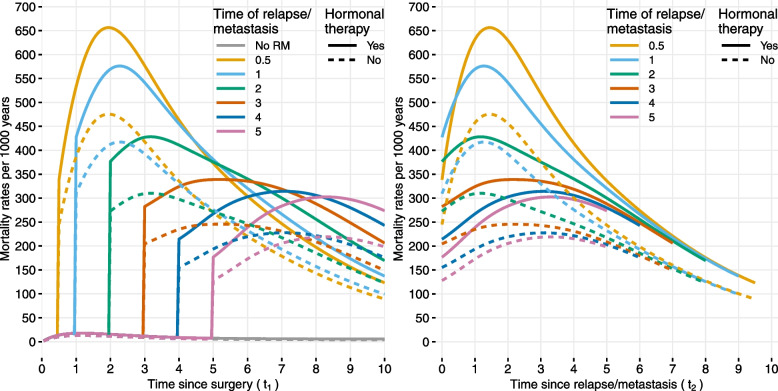
Based on Rotterdam Breast Cancer data, estimated mortality rates per 1000 person-years for breast cancer patients who were aged 50 at primary surgery and were treated with hormonal therapy (solid lines) and without hormonal therapy (dotted lines). **Left panel** shows the mortality rates over time since surgery (time-scale $t_1$) for patients having relapse or metastasis (RM) at different time-points since surgery as well as for non-RM patients. **Right panel** shows the mortality rates for RM patients along the time since RM (time-scale $t_2$)

The FPM and Poisson approaches yielded almost identical results; coefficients and 95% confidence intervals (CI) are displayed in Table 1. The spline coefficients are not interpretable on their own but they are used to predict the shape of the hazard surface at different covariate values. For example, Fig. 1 displays two panels for viewing the estimated mortality rates from the FPM. The left panel shows the estimated mortality rates per 1000 person-years along time since surgery (time-scale $t_1$) for patients who did not have RM and for patients who experienced RM at 0.5, 1, 2, 3, 4, 5 years post surgery, respectively. The jumps from “No RM” at these time-points post-surgery into the RM state are represented by the vertical lines. The right panel of Fig. 1 provides an alternative view, along the second time-scale, time since RM (time-scale $t_2 = t_1-r$, with *r* as offset as time of RM post surgery). From both panels, as from Supplementary Fig. 1, we see that the mortality rates decrease with both time since surgery and with time since RM. Patients treated without the hormonal therapy also have lower mortality rates than patients who received hormonal therapy. The purpose of this article is to demonstrate the methods, therefore it is not recommended to interpret the results in the clinical sense. Nevertheless, the high mortality among patients treated with hormonal therapy can be due to a more severe disease for this group of patients. As in Fig. 1, in subsequent figures, we displayed results from the FPM only, as there was a complete overlap between the two methods of estimated mortality rates and survival proportions. Furthermore, for demonstration purposes, all the predicted values were calculated for patients who had primary surgery at age 50.

**Table 1:** Coefficients and 95% confidence intervals from the FPM and Poisson models of the mortality rates with two time-scales (t1 as time since surgery, and t2 as time since relapse or metastasis (RM)) and a time-varying indicator for the second time-scale (Indicator for RM) for Rotterdam Breast Cancer patients. Note: the spline coefficients are not interpretable on their own but they are used for making smooth predictions of the mortality rates

	FPM	Poisson
Indicator for RM	3.1891 (2.9314, 3.4468)	3.1937 (2.9362, 3.4512)
Age spline 1	-0.0578 (-0.1092, -0.0063)	-0.0578 (-0.1092, -0.0063)
Age spline 2	-0.0005 (-0.0009, 0)	-0.0005 (-0.0009, 0)
Age spline 3	0.0006 (-0.0002, 0.0014)	0.0006 (-0.0002, 0.0014)
Age spline 4	-0.0001 (-0.0008, 0.0005)	-0.0001 (-0.0008, 0.0005)
Age spline 5	-0.0001 (-0.0004, 0.0002)	-0.0001 (-0.0004, 0.0002)
Indicator for hormonal therapy	0.3224 (0.1439, 0.5009)	0.3221 (0.1436, 0.5006)
log(t1) spline 1	0.8853 (0.4266, 1.344)	0.8802 (0.4226, 1.3379)
log(t1) spline 2	0.7396 (0.3272, 1.1519)	0.7364 (0.3243, 1.1486)
log(t1) spline 3	-1.0513 (-1.7461, -0.3565)	-1.0467 (-1.7412, -0.3521)
t2 spline 1	0.4519 (0.1646, 0.7392)	0.4465 (0.1594, 0.7336)
t2 spline 2	0.0562 (-0.0555, 0.1679)	0.0546 (-0.0571, 0.1662)
t2 spline 3	-0.0153 (-0.0711, 0.0404)	-0.0146 (-0.0703, 0.0411)
Intercept	-4.0529 (-4.3845, -3.7213)	-4.0549 (-4.3864, -3.7234)

Supplementary Fig. 1 shows the estimated mortality rates per 1000 person-years over both time-scales in three-dimensional view for patients who had primary surgery at age 50, received hormonal therapy and experienced RM. It can be seen that the peak of the surface with grey and orange belts (mortality rates higher than 600) is concentrated over the quadrant of one to three years since surgery and one to three years since RM. The mortality rates surface becomes more “shallow” with progression of time on both time-scales similar to what is observed in clinical practice [23].

**Fig. 2:**
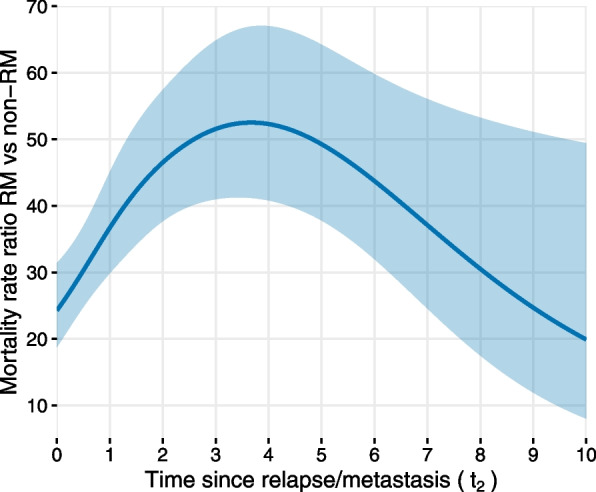
Estimated mortality rate ratio and 95% confidence interval for breast cancer patients in the Rotterdam Breast Cancer data, experiencing relapse or metastasis (RM) after primary surgery versus patients without RM, over time since RM

We can also assess the time-varying effect of experiencing RM on mortality rates compared to not having RM given the same time since surgery, same age at surgery and same treatment with or without hormonal therapy. This is represented by mortality rate ratio with 95% CI over time since RM in Fig. 2. From Fig. 2 we observe that the relative effect of having RM increases rapidly with time since RM: from rate ratio 24.27 (95% CI: 18.75, 31.4) at time 0 since RM to rate ratio of 52.51 (95% CI: 41.2, 66.93) at 3.65 years since RM, followed by a rapid decline reaching 19.91 (95% CI: 8.02, 49.41) at 10 years since RM. To clarify further this comparison, the mortality rate for individuals at 10 years since RM is 19.91 times the mortality rate for individuals who never experience RM given these individuals have the same time since surgery, the same age and therapy. The model assumes that the mortality rate ratio at a certain time since RM (i.e. at certain values of $t_2$=$t_1-r$) is the same irrespective of time since surgery (i.e. $t_1$) and time of RM (i.e. *r*) as long as the comparison is made between individuals having the same time since surgery. This means that we get the same mortality rate ratio for the effect of RM in comparison to no RM when, for example, $t_1=2$, $r=1$, and $t_1=3$, $r=2$, as both comparisons are at one year after RM. This comes from the fact that the model does not include an interaction term between RM and $t_1$, nor does it include an interaction between $t_1$ and $t_2$ time-scales. However, with more complex models that include interactions between time-scales as well as time-dependent effects of the covariates, the graphical representation of the mortality rate ratio would have to be displayed for different values of both time-scales.

**Fig. 3:**
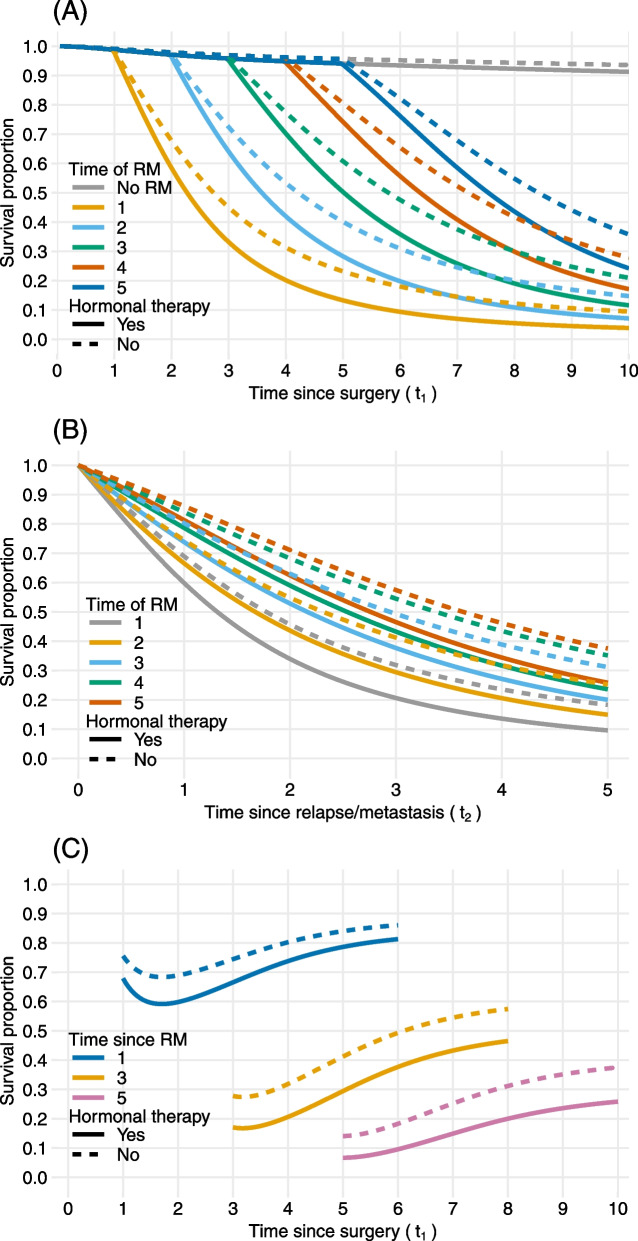
Predicted survival proportions for breast cancer patients in the Rotterdam Breast Cancer data, who were aged 50 at primary surgery and were treated with hormonal therapy (solid lines) and without hormonal therapy (dotted lines). **Panel (A)** shows the survival proportions over time since surgery (time-scale $t_1$) for patients having relapse or metastasis (RM) at different time-points since surgery as well as for non-RM patients. **Panel (B)** shows the survival proportions since RM (time-scale $t_2$) for patients having RM at different time-points. **Panel (C)** shows 1-, 3- and 5-year survival proportions since RM across time since surgery

In addition to the mortality rates, we can also obtain survival proportions for different sub-groups along both time-scales. The 95% CI (not shown) were computed using the bias-corrected percentile bootstrap method with 1000 samples. Figure 3 provides examples of predicted survival probabilities on either of the time-scales. For example, panel (A) shows smooth predicted survival curves over time since surgery for patients without RM and for patients with RM at 1, 2, 3, 4, 5 years post surgery. Panel (B), on the other hand, depicts the survival proportions over time since RM. Both panels supplement each other and aid in comparison of the sub-groups. In addition to the conventional representations of predicted survival in panels (A) and (B), it can be of interest to assess the survival proportions chosen for specific time-points since RM and time-points since surgery. Panel (C) displays the probability of surviving 1, 3 and 5 years after RM across different time points since surgery. It can be seen that the survival proportions are higher for patients with longer time since surgery given the same time since RM. For example, the probability to survive three years after RM for patients on hormonal therapy is 0.206 (95% CI: 0.142, 0.276) and 0.465 (95% CI: 0.394, 0.544) at four and eight years since surgery, respectively.

### Matched cohort

In a matched cohort, when individuals with a certain disease or characteristic are matched to population controls, or comparators without the characteristics, using matching variables such as age, sex and calendar year, then attained age is often the most relevant time-scale. However, time since diagnosis can also be of importance for the exposed individuals, but is not relevant for the matched comparators in the cohort. We explore this type of scenario by analysing matched cohort data of Swedish patients with myeloproliferative neoplasms (MPN). Our objective was to model mortality rates and estimate survival proportions for the MPN cases compared to population comparators over the time-scales attained age and time since diagnosis.

A detailed description of the disease and patient characteristics of the MPN matched cohort are provided elsewhere [24]. Briefly, each MPN patient at age 18 years or older was matched to four individuals from the Swedish general population based on sex, age and calendar year of diagnosis during 1987 - 2009. Furthermore, the first 30 days of follow-up were excluded to avoid surveillance bias. In total the cohort consisted of 9,164 MPN cases and 35,763 matched controls with follow-up until death or censoring on December 31, 2010 or at maximum of 10 years of follow-up. In total 5,108 deaths were observed among MPN subjects, and 11,677 among non-MPN subjects (overall mortality rates were 91 and 39 per 1000 person-years, respectively).

Similar to the previous case-study, using the FPM and Poisson methods we fitted the following hazard model,

7 $$\begin{aligned} \log (h(t_1, t_1-a_0; \boldsymbol{\gamma }_{g}, \boldsymbol{k}_{g}, \boldsymbol{\gamma }_{v}, \boldsymbol{k}_{v}, \boldsymbol{\beta }))= & {} g_0(t_1; \boldsymbol{\gamma }_{g}, \boldsymbol{k}_{g}) + v_0(t_1-a_0; \boldsymbol{\gamma }_{v}, \boldsymbol{k}_{v}) \cdot I_{MPN}\nonumber \\ &+ \beta _{MPN} \cdot I_{MPN} + \beta _{sex} \cdot I_{sex}, \end{aligned}$$

where $t_1$ is attained age (reference time-scale), $t_1-a_0$ is time since diagnosis (second time-scale) with $a_0$ as age at MPN diagnosis, $I_{MPN}$ is the indicator for the MPN cases, and $I_{sex}$ is the indicator for sex (0 = Men, 1 = Women). Even though this model might not be the most relevant, in terms of covariates included, it is useful for demonstration purposes. Furthermore, it has been shown that there is no need to include all matching variables in the model in the analysis of matched cohort data if there is no additional confounding [25]. In both FPM and Poisson methods, we chose five knots for both spline functions $g_0, v_0$, equally placed over the distribution of event-times, where the first knot and the last knot are placed at the first and last event times, respectively. Additionally, for the Poisson method, we split the person-time by two-day intervals, and used mid-points of the intervals when creating the splines.

#### Graphical representations of results for matched cohort

As in the previous case-study, the coefficients and 95% CI displayed in Table 2 from the FPM and Poisson approaches are almost identical. The predicted mortality rates and survival proportions were the same from both methods, hence the figures that follow, represent results for attained ages 60-95 from the FPM method only.

**Table 2:** Coefficients and 95% confidence intervals from the FPM and Poisson models of the mortality rates with two time-scales (t1 as attained age, and t2 as time since MPN diagnosis) for cohort of Swedish MPN patients diagnosed during 1987-2009 and matched comparators. Note: the spline coefficients are not interpretable on their own but they are used for making smooth predictions of the mortality rates

	FPM	Poisson
Indicator for MPN	1.5563 (1.4543, 1.6583)	1.5620 (1.4602, 1.6639)
Indicator for sex (0 = Men, 1 = Women)	-0.3273 (-0.3623, -0.2923)	-0.3272 (-0.3622, -0.2922)
t1 spline 1	0.0657 (0.0508, 0.0805)	0.0657 (0.0508, 0.0805)
t1 spline 2	0.0001 (-0.0001, 0.0002)	0.0001 (-0.0001, 0.0002)
t1 spline 3	-0.0006 (-0.0010, -0.0003)	-0.0006 (-0.0010, -0.0003)
t1 spline 4	0.0007 (0.0004, 0.0010)	0.0007 (0.0004, 0.0010)
t2 spline 1	-0.4661 (-0.6089, -0.3234)	-0.4740 (-0.6167, -0.3314)
t2 spline 2	-0.0821 (-0.1315, -0.0327)	-0.0842 (-0.1336, -0.0348)
t2 spline 3	0.0344 (0.0045, 0.0644)	0.0355 (0.0055, 0.0654)
t2 spline 4	-0.0028 (-0.0113, 0.0058)	-0.0029 (-0.0114, 0.0057)
Intercept	-8.8858 (-9.6864, -8.0853)	-8.8859 (-9.6864, -8.0854)

**Fig. 4:**
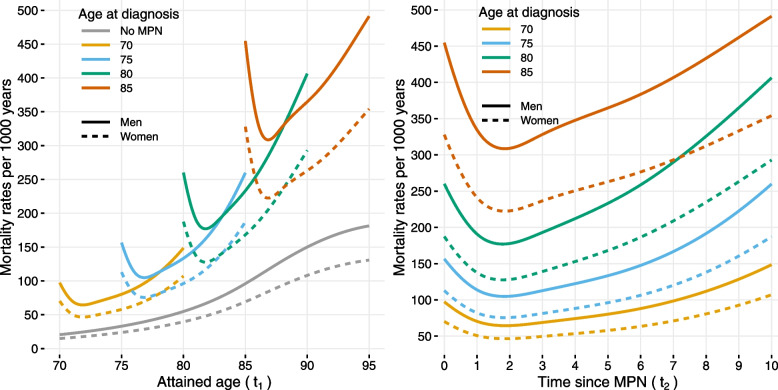
Estimated mortality rates per 1000 person-years for Swedish MPN patients and matched comparators where solid lines are for men and dotted lines are for women. **Left panel** shows the mortality rates over attained age (time-scale $t_1$) for patients with different ages at diagnosis and for comparators. **Right panel** shows the mortality rates for MPN patients along the time since diagnosis (time-scale $t_2$)

From both panels of Fig. 4, it can be seen that for the same attained age, the mortality for male MPN patients is much higher right after diagnosis than after 10 years of having the disease. For example, among men at age 80 right after the diagnosis, the mortality rate is 260 per 1000 person-years, whereas for 80 year-olds that were diagnosed 10 years ago the rate is 149 per 1000 person-years. However, this mortality rate is still higher than the mortality rate for 80 year-olds who were diagnosed five years ago (133 per 1000 person-years). Additionally, the mortality rates surface per 1000 person-years in Supplementary Fig. 2 shows a characteristic steep increase in the mortality rates with progression of time on both time-scales, attained age and time since diagnosis.

**Fig. 5:**
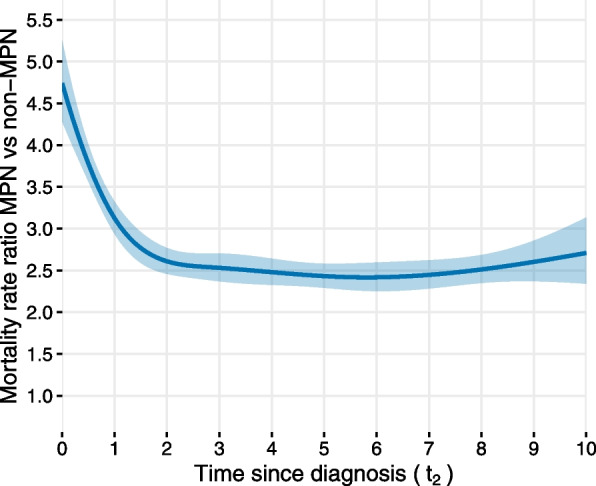
Estimated mortality rate ratio and 95% confidence interval over time since diagnosis for Swedish MPN patients in comparison to matched comparators without MPN

After an initial decline in the first two years since diagnosis, given the same attained age and sex, the mortality rate ratio is shown to be averaging at 2.51 (95% CI: 2.33, 2.7) for MPN cases relative to non-MPN matched controls over time since diagnosis (Fig. 5). A steep initial decline in mortality after diagnosis has been observed also for other diseases, for example, diabetes [26].

**Fig. 6:**
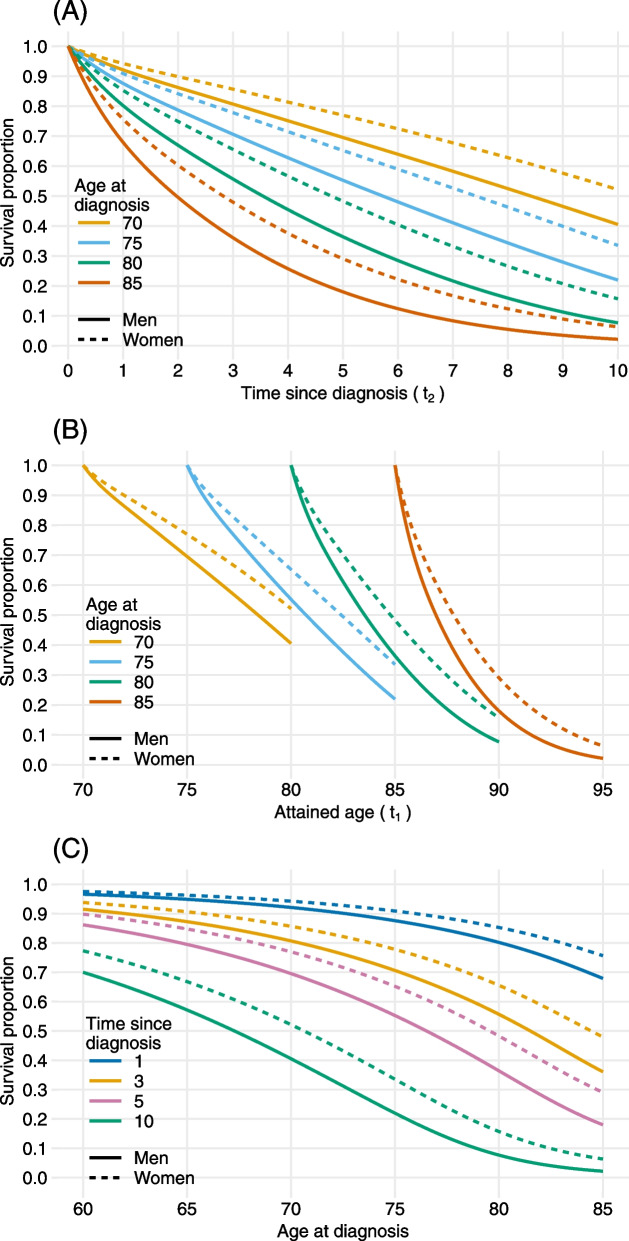
Predicted survival proportions for Swedish MPN patients where solid lines are for men and dotted lines are for women. **Panel (A)** shows the survival proportions over time since diagnosis (time-scale $t_2$). **Panel (B)** shows the survival proportions over attained age (time-scale $t_1$) for different ages at diagnosis. **Panel (C)** shows 1-, 3-, 5- and 10-year survival proportions across age at diagnosis

Different graphical views of survival proportions are shown in Fig. 6. Panels (A) and (B) provide two alternative displays of the same survival proportions for patients with ages at diagnosis 70, 75, 80, 85 over time-scales time since diagnosis and attained age, respectively. As expected, younger patients have better survival as well as women in all age groups. In panel (C), we have a cross-sectional view of panel (B), where 1-, 3-, 5-, 10-year survival proportions are plotted with respect to age at diagnosis. The dramatic decrease in survival for older age groups given the same lengths of time since diagnosis is more apparent in this figure. For example, the 5-year overall survival is 81.07% (95% CI: 80.09, 81.99) and 58.49% (95% CI: 57.09, 59.8) for a male patient diagnosed at age 64 and 74, respectively.

## Discussion

There are situations when it is necessary to include multiple time-scales in a hazard model. For example, both woman’s age and time since first childbirth have a simultaneous impact on breast cancer incidence [27] and mortality among individuals with dementia is dependent on time since onset and attained age [28], and for chronic diseases, such as diabetes mellitus, rates of complications are dependent on person’s age and duration of the disease [26]. The study design may dictate which time-scales to include in the hazard model. For example, in the illness-death framework by allowing non-Markov assumption, the baseline hazard can take into account both time from the initial state as well as time from the intermediate state. And, in the matched cohort design, it may be required that the hazard model includes additional time-scales specific to the subgroups of the cohort. A recent study has also demonstrated how multiple time-scales can be incorporated within the relative survival framework in a multistate setting [29].

An established method, such as the Poisson GLM, is a powerful tool when it comes to modeling the hazard rates with multiple time-scales. However, users are required to split the dataset along the chosen time-scales and make an unrealistic assumption of piecewise constant hazard rates within the time-intervals. Furthermore, with longer follow-up times, and larger volumes of data, it can be computationally challenging to model with a large dataset containing finely split person-time. For certain studies it might be sufficient to split along one time-scale and keep track of the time-intervals on other time-scales, but there can be situations when it is necessary to split along two or more time-scales simultaneously, and therefore, increasing the computational burden, as well as running a higher risk of making errors when it comes to splitting the person-time along multiple time-scales.

In this study we aimed to introduce and demonstrate the flexible parametric survival models that can capture complex shapes of the baseline hazard function with multiple time-scales given other time-scale(s) is(are) expressed in terms of the reference time-scale and offsets or times of origin. With the FPM, users avoid time-splitting of the dataset as well as making an assumption of piecewise constant hazard rates. This assumption can be relaxed in a Poisson model by the use of splines or other smoothing techniques, but that requires splitting the data in more intervals for the time-scales to be treated as continuous variables. We compared the FPM and the Poisson models with time-splitting in two-day intervals in both case-studies and using splines for modelling the time-scales, showing that the estimates from the fitted models were nearly identical. However, in the figures we displayed the predicted hazard rates and survival proportions from the FPM only, as there was a complete overlap with the Poisson method. We also compared the results from the Poisson models with time-splitting in one-month intervals (results not shown). The estimated coefficients were close to the coefficients from the FPM method but not as close as from the model with data split in two-day intervals. Also the estimated mortality rates did not always overlap within given time-frames. However, the survival proportions were seen to overlap.

We also demonstrated different ways of presenting the results graphically, that can help users understand the disease better. Plotting the hazard rates and survival probabilities with respect to each time-scale separately for different time-points from the other time-scale can be also helpful to answer different research questions in regards to the disease of interest. The implementation of all the analyses in Stata for the model with time-varying covariate is shown in the Supplementary material.

There are limitations in this study. The confidence intervals for survival proportions were obtained using the bias-corrected percentile bootstrap as estimation of survival with confidence intervals has not been implemented as part of the software package. Future work should implement easier estimation of confidence intervals with the use of the delta method [30]. Another limitation that is inherent to the FPM, is choosing knots for the spline functions. However, according to Syriopoulou et al. (2019) [31], as long as there are not too few knots, the results are robust to different choices of knots. We also did not address the question of model fitting in this study. This is the most challenging part of fitting models with multiple time-scales as there are so many aspects to consider in addition to choosing covariates, such as determining which time-dependent effects on which time-scales to include, or whether there are interactions between the time-scales. With each time-scale comes an additional dimension of all the challenges that users face with one-time-scale models.

In conclusion, by using the FPM, users can avoid splitting the survival data into intervals and avoid making an assumption of piecewise constant hazard rates within the time-intervals, while obtaining all the necessary estimates for inference and the graphical representation of the estimated hazard rates and probabilities. The examples shown were used to highlight the importance of fitting models with multiple time-scales, and how results from these complex models can be graphically presented.

## Supplementary Information

**Additional file 1.** Stata .do files containing Stata code for the analysis of the model with time-varying covariates.

**Additional file 2: Supplementary Figure 1.**

**Additional file 3: Supplementary Figure 2.**

## Data Availability

Analysis data as well as Stata code for the model with a time-varying covariate are provided in the Supplementary material.

## Abbreviations

FPM: Flexible parametric survival model
H: Proportional hazards
RM: Relapse or metastasis
CI: Confidence intervals
MPN: Myeloproliferative neoplasm
GLM: Generalised linear model

## References

[1] Efron B (1988). Logistic Regression, Survival Analysis, and the Kaplan-Meier Curve. J Am Stat Assoc..

[2] Cox DR (1972). Regression Models and Life Tables. J R Stat Soc.

[3] Royston P, Altman DG (1994). Regression Using Fractional Polynomials of Continuous Covariates: Parsimonious Parametric Modelling. J R Stat Soc Ser C (Appl Stat)..

[4] Herndon JE, Harrell FE (1990). The restricted cubic spline hazard model. Commun Stat Theory Methods..

[5] Royston P, Parmar MKB (2002). Flexible Parametric Proportional-Hazards and Proportional-Odds Models for Censored Survival Data, with Application to Prognostic Modelling and Estimation of Treatment Effects. Statistics in Medicine..

[6] Lambert PC, Royston P (2009). Further Development of Flexible Parametric Models for Survival Analysis. Stata J..

[7] Farewell VT, Cox DR (1979). A Note on Multiple Time Scales in Life Testing. J R Stat Soc Ser C (Appl Stat)..

[8] Oakes D (1995). Multiple Time Scales in Survival Analysis. Lifetime Data Anal..

[9] Korn EL, Graubard BI, Midthune D (1997). Time-to-Event Analysis of Longitudinal Follow-up of a Survey: Choice of the Time-Scale. Am J Epidemiol..

[10] Thiébaut ACM, Bénichou J (2004). Choice of Time-Scale in Cox’s Model Analysis of Epidemiologic Cohort Data: A Simulation Study. Stat Med..

[11] Pencina MJ, Larson MG, D’Agostino RB (2007). Choice of Time Scale and Its Effect on Significance of Predictors in Longitudinal Studies. Stat Med..

[12] Chalise P, Chicken E, McGee D (2013). Performance and Prediction for Varying Survival Time Scales. Commun Stat Simul Comput..

[13] Holford TR (1983). The Estimation of Age, Period and Cohort Effects for Vital Rates. Biometrics..

[14] Keiding N (1990). Statistical Inference in the Lexis Diagram. Philos Trans R Soc Lond Ser A Phys Eng Sci..

[15] Efron B (2002). The Two-Way Proportional Hazards Model. J R Stat Soc Ser B (Stat Methodol)..

[16] Iacobelli S, Carstensen B (2013). Multiple Time Scales in Multi-State Models. Stat Med..

[17] Crowther MJ, Lambert PC (2014). A General Framework for Parametric Survival Analysis. Stat Med..

[18] Bower H, Crowther MJ, Lambert PC (2016). Strcs: A Command for Fitting Flexible Parametric Survival Models on the Log-hazard Scale. The Stata Journal: Promoting communications on statistics and Stata..

[19] Bower H, Crowther MJ, Rutherford MJ, Andersson TML, Clements M, Liu XR, et al. Capturing simple and complex time-dependent effects using flexible parametric survival models: A simulation study. Commun Stat Simul Comput. 2019;1–17. 10.1080/03610918.2019.1634201.

[20] Carstensen B (2007). Age-Period-Cohort Models for the Lexis Diagram. Stat Med..

[21] Foekens JA, Peters HA, Look MP, Portengen H, Schmitt M, Kramer MD (2000). The Urokinase System of Plasminogen Activation and Prognosis in 2780 Breast Cancer Patients. Cancer Res..

[22] Sauerbrei W, Royston P, Look M (2007). A New Proposal for Multivariable Modelling of Time-Varying Effects in Survival Data Based on Fractional Polynomial Time-Transformation. Biom J..

[23] Dent R, Valentini A, Hanna W, Rawlinson E, Rakovitch E, Sun P (2014). Factors Associated with Breast Cancer Mortality after Local Recurrence. Curr Oncol..

[24] Hultcrantz M, Björkholm M, Dickman PW, Landgren O, Derolf ÅR, Kristinsson SY (2018). Risk for Arterial and Venous Thrombosis in Patients With Myeloproliferative Neoplasms. Ann Intern Med..

[25] Sjölander A, Greenland S (2013). Ignoring the Matching Variables in Cohort Studies – When Is It Valid and Why?. Stat Med..

[26] Huo L, Magliano DJ, Rancière F, Harding JL, Nanayakkara N, Shaw JE (2018). Impact of Age at Diagnosis and Duration of Type 2 Diabetes on Mortality in Australia 1997–2011. Diabetologia..

[27] Albrektsen G, Heuch I, Hansen S, Kvåle G (2005). Breast Cancer Risk by Age at Birth, Time since Birth and Time Intervals between Births: Exploring Interaction Effects. Br J Cancer..

[28] Commenges D, Joly P, Letenneur L, Dartigues J (2004). Incidence and Mortality of Alzheimer’s Disease or Dementia Using an Illness-Death Model. Stat Med..

[29] Weibull CE, Lambert PC, Eloranta S, Andersson TML, Dickman PW, Crowther MJ (2021). A Multistate Model Incorporating Estimation of Excess Hazards and Multiple Time Scales. Stat Med..

[30] Hosmer DW, Lemeshow S, May S. Appendix 1: The Delta Method. In: Applied Survival Analysis. Wiley; 2008. p. 355–358. 10.1002/9780470258019.app1.

[31] Syriopoulou E, Mozumder SI, Rutherford MJ, Lambert PC (2019). Robustness of Individual and Marginal Model-Based Estimates: A Sensitivity Analysis of Flexible Parametric Models. Cancer Epidemiol..

