# Supplementary material for: Modelling multiple time-scales with flexible parametric survival models
Source: BMC Med Res Methodol. 2022 Nov 9;22:290. doi: 10.1186/s12874-022-01773-9 (PMC9644623; doi:10.1186/s12874-022-01773-9)
Supplement: Supplementary file 2 — Additional file 2: Supplementary Figure 1. [file 12874_2022_1773_MOESM2_ESM.docx]

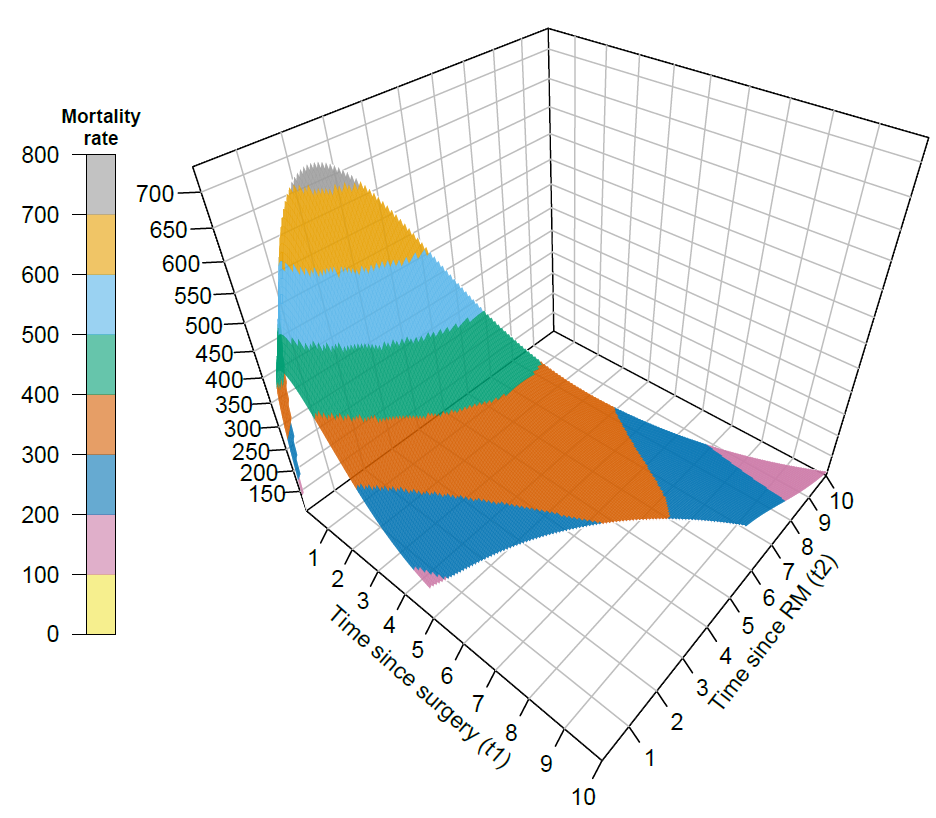


Supplementary Figure 1: Estimated mortality rates surface per 1000 person-years over two time-scales for breast cancer patients in the Rotterdam Breast Cancer data, who were aged 50 at primary surgery, received hormonal therapy and experienced relapse or metastasis (RM).
