# Supplementary material for: Modelling multiple time-scales with flexible parametric survival models
Source: BMC Med Res Methodol. 2022 Nov 9;22:290. doi: 10.1186/s12874-022-01773-9 (PMC9644623; doi:10.1186/s12874-022-01773-9)
Supplement: Supplementary file 3 — Additional file 3: Supplementary Figure 2. [file 12874_2022_1773_MOESM3_ESM.docx]

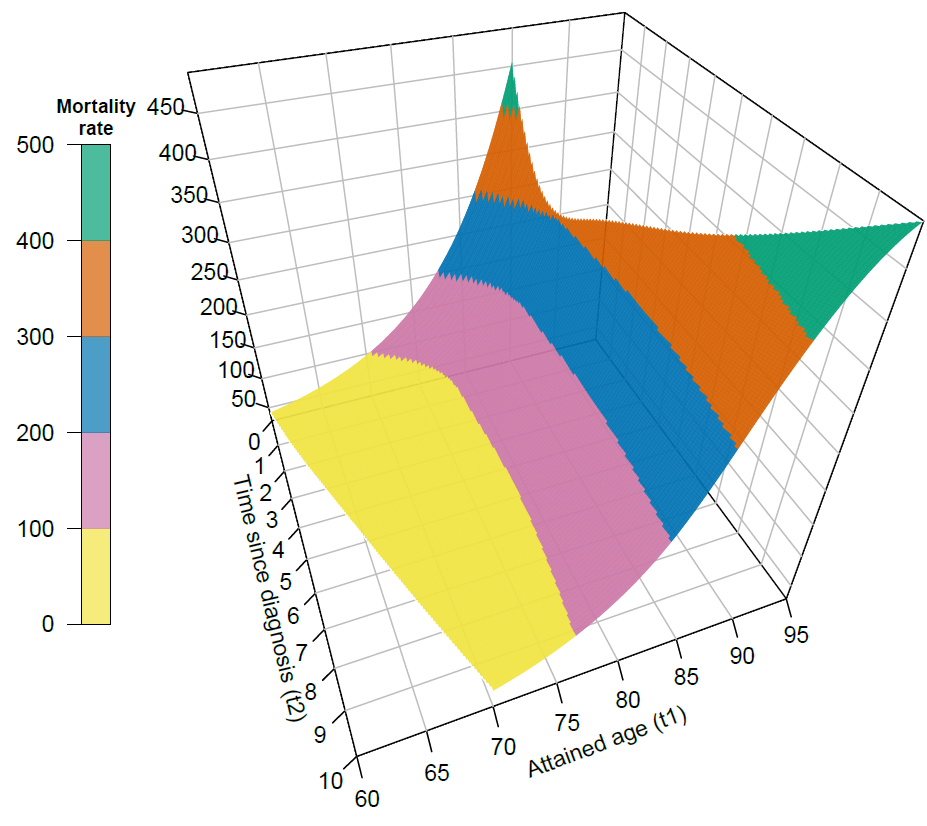
 Supplementary Figure 2: Estimated mortality rates surface per 1000 person-years over two time-scales for male MPN patients in Sweden.
